# Zinc Metallochaperones as Mutant p53 Reactivators: A New Paradigm in Cancer Therapeutics

**DOI:** 10.3390/cancers10060166

**Published:** 2018-05-29

**Authors:** Samuel Kogan, Darren R. Carpizo

**Affiliations:** 1 Department of Surgery, Rutgers Robert Wood Johnson Medical School, New Brunswick, NJ 08901, USA; samkogan@cinj.rutgers.edu; 2 Rutgers Cancer Institute of New Jersey, 195 Little Albany Street, New Brunswick, NJ 08901, USA; 3 Department of Pharmacology, Rutgers University, Piscataway, NJ 08854, USA; 4 Z53 Therapeutics, Inc., Holmdel, NJ 07733, USA

**Keywords:** zinc metallochaperones, mutant p53, on/off switch mechanism, zinc homeostasis, pancreatic cancer

## Abstract

Restoration of wild-type structure and function to mutant p53 with a small molecule (hereafter referred to as “reactivating” mutant p53) is one of the holy grails in cancer therapeutics. The majority of *TP53* mutations are missense which generate a defective protein that is targetable. We are currently developing a new class of mutant p53 reactivators called zinc metallochaperones (ZMCs) and, here, we review our current understanding of them. The p53 protein requires the binding of a single zinc ion, coordinated by four amino acids in the DNA binding domain, for proper structure and function. Loss of the wild-type structure by impairing zinc binding is a common mechanism of inactivating p53. ZMCs reactivate mutant p53 using a novel two-part mechanism that involves restoring the wild-type structure by reestablishing zinc binding and activating p53 through post-translational modifications induced by cellular reactive oxygen species (ROS). The former causes a wild-type conformation change, the later induces a p53-mediated apoptotic program to kill the cancer cell. ZMCs are small molecule metal ion chelators that bind zinc and other divalent metal ions strong enough to remove zinc from serum albumin, but weak enough to donate it to mutant p53. Recently we have extended our understanding of the mechanism of ZMCs to the role of cells’ response to this zinc surge. We found that cellular zinc homeostatic mechanisms, which normally function to maintain free intracellular zinc levels in the picomolar range, are induced by ZMCs. By normalizing zinc levels, they function as an OFF switch to ZMCs because zinc levels are no longer sufficiently high to maintain a wild-type structure. This on/off switch leads to a transient nature to the mechanism of ZMCs in which mutant p53 activity comes on in a few hours and then is turned off. This finding has important implications for the translation of ZMCs to the clinic because it indicates that ZMC concentrations need not be maintained at high levels for their activity. Indeed, we found that short exposures (as little as 15 min) were adequate to observe the mutant p53 reactivating activity. This switch mechanism imparts an advantage over other targeted therapeutics in that efficacy can be accomplished with minimal exposure which minimizes toxicity and maximizes the therapeutic window. This on/off switch mechanism is unique in targeted cancer therapeutics and will impact the design of human clinical trials.

## 1. Introduction

### 1.1. The Multi-Functional Role of p53

The *TP53* gene is the most frequently mutated gene in all of human cancer, with at least 50% of cancers harboring a mutation, which in part explains why the p53 protein is one of the most intensively studied transcription factors [1]. In addition to its role as a potent tumor suppressor, p53 has come to be understood as a critical responder to cellular stress of a wide variety of etiologies, including DNA damage, oncogene activation, and reactive oxygen species [2]. Under normal cellular conditions, p53 levels are under very tight control due to a negative feedback loop involving MDM2, an E3-ubiquitin ligase and p53-target gene, which labels p53 for proteasomal degradation. The p53 protein is primarily regulated at the post-translational level, through post-translational modifications (PTMs) of p53 including, but not limited to, phosphorylation, acetylation, methylation, di-methylation, mono-ubiquitination, poly-ubiquitination, SUMOylation, and ADP-ribosylation [3]. These modifications regulate p53 function by affecting its stabilization, as well as its binding to the promoters of different target genes.

#### Over Three Decades the Spectrum of Cellular Functions of p53 Has Grown Substantially 

The p53 protein controls a number of cellular pathways through regulation of downstream p53-target genes. Some of the principle pathways associated with the tumor suppressive activity of p53 include cell-cycle arrest, cellular senescence, and apoptosis. Additional roles of p53 include its regulation of DNA repair, cellular differentiation, metabolism, fertility, and stem cell renewal [4]. Evidence continues to mount for the role of p53 in tumor suppression beyond the classical cell cycle arrest, apoptosis, and senescence. Wei Gu’s group generated mutant mice in which the three lysine residues of the DNA-binding domain were replaced with arginine [5]. These substitutions prevented acetylation of the DNA-binding domain and abrogated the ability of p53 to induce apoptosis, cell-cycle arrest, and senescence in vivo. Interestingly, these mutant mice did not succumb to early-onset spontaneous tumors that are commonly observed in p53-null mice, suggesting that apoptosis, cell-cycle arrest, and senescence are not the only p53-dependent mediators of tumor suppression. The mutant p53 protein expressed in these mice was still able to regulate the expression of metabolic p53-target genes, proposing a role of metabolism regulation in tumor suppression [5]. 

### 1.2. p53 Mutations and Cancer: A Unique Tumor Suppressor 

The p53 protein is composed of three principle domains: the N-terminal domain containing the transcriptional activation domain (TAD, residues 1–94); the DNA-binding domain (DBD, residues 94–292); and the C-terminal tetramerization domain (TAT, residues 292–393) [6]. The majority of *TP53* mutations (75%) are missense mutations in which a defective protein is made, which differs from other tumor suppressors such as *RB1*, *APC*, and *PTEN* which typically are null mutations [7,8]. Ninety-five percent of the missense mutations occur within the DNA-binding core domain (DBD) [8]. Many of the missense mutations of p53 increase free energy of the protein, resulting in destabilization of its structure [9]. Interestingly there are a group of missense mutations that occur far more frequently than others and are known as “hot spot” residues, which include R248, R273, R175, G245, R249, and R282 [10]. It is currently unclear as to why these particular codon sites are selected for during tumor progression.

Missense mutations in p53 can either impair or completely abolish the activity of wild-type p53. Since p53 functions as a tetramer, mutant p53 may also function as a dominant negative inhibitor over any remaining wild-type p53 [11]. The most accepted explanation for why missense mutations are selected for during tumor progression comes from the concept that these missense mutant proteins are functional and impart a more favorable biology to the tumor in a number of ways. This concept is generally known as the mutant p53 gain of function (GOF) phenotype. This concept was first suggested when mutant p53 was introduced into p53 null cells and new phenotypes were observed (enhanced tumorigenic potential in nude mice and enhanced plating efficiency in agar cell culture) [12]. This concept is further supported by the observation that patients carrying a germline *TP53* missense mutation for a mutant p53 protein develop cancers significantly earlier than patients carrying *TP53* null mutations [13]. Similarly, mice expressing mutant p53 develop cancers that are more metastatic and aggressive than the cancers observed in p53 null or wild-type mice [11]. While the majority of p53 mutations occur in the “hot spot” residues, mutations in nearly every codon of the DNA-binding domain of p53 have been observed in cancer. Accumulating evidence suggests that the wide range of p53 mutations have varied consequences with respect to loss of wild-type activity, ability to inhibit remaining wild-type protein function, and acquisition of GOF [11]. 

Another GOF property of mutant p53 is the upregulation of chromatin regulatory genes, leading to genome-wide increases in methylation and acetylation [14]. Specifically, mutant p53 proteins drive the expression of the methyltransferases *KMT2A* (*MLL1*) and *MKT2D* (*MLL2*) and acetyltransferase *KAT6A* (*MOZ* or *MYST3*). Patient-derived tumors expressing GOF p53 mutants show upregulation of *MLL1*, *MLL2*, and *MOZ*, whereas wild-type or p53 null tumors did not. These genetic changes contribute to cancer cell proliferation and can be attenuated by knockdown of MML1 or pharmacologic inhibition of the MLL1 methyltransferase complex [14]. 

Germline mutations in *TP53* are associated with Li-Fraumeni syndrome, an inherited condition of increased predisposition for developing several types of cancers [15]. Interestingly, among Brazilian patients with Li-Fraumeni syndrome, there appears to be a strong selection for the R337H missense mutation [16,17]. Unlike the hot spot mutations which all cluster within the DBD, this mutation occurs in the oligomerization domain and impairs dimerization in a pH-dependent manner [18]. 

In a majority of tumors bearing wild-type p53, alternative mechanisms exist to inactivate the tumor suppressor. For example, many DNA oncoviruses produce proteins that inactivate p53 including: SV40 large T-antigen, adenovirus E1B-55-kDa protein, and the E6 oncoprotein of human papilloma virus (HPV) types 16 and 18 [19]. Additionally, tumors can abrogate the activity of wild-type p53 by upregulation of negative regulators of p53. Mdm2 and its homolog Mdmx have been shown to be upregulated in some tumors [20]. 

Mutations in p53 impair consensus-DNA binding and proper folding of the protein. One of the consequences of this is loss of MDM2-mediated degradation of the mutant protein. As a result, mutant p53 levels are very high in the cancer cell, and thus theoretically, can serve as a pharmacological target. Restoring wild-type function to these mutant proteins using a small molecule has been the subject of intense investigation in developmental therapeutics. Numerous approaches have been described, many of which have been discovered in the last ten years and are summarized in several reviews [2,21,22]. 

### 1.3. Classes of p53 Mutations

Mutations in p53 can broadly be categorized as either DNA-contact, structural/stability, or zinc-binding [23]. DNA-contact mutations, like p53-R273H, have amino acid substitutions in residues that directly interact with DNA. While DNA-contact mutations do not significantly alter p53 structure [24], they can affect protein thermostability as measured by melting temperature [25]. Contrastingly, structural or stability mutants often occur in the hydrophobic ß-sheet and substitute amino acids that impair folding of the core domain [9,24]. Structural mutants more strongly reduce thermostability of the protein and more extensively distort the protein structure [25]. 

Zinc-binding mutations are classified based on their proximity to the loops involved in coordination of a single zinc ion [26]. The zinc ion is coordinated by four amino acids (Cys-176, His-179, Cys-238, and Cys-242). The L2 and L3 loops of the DBD are stabilized by this zinc ion [6]. Mutations at any of these four amino acids impair zinc binding and, thus, disrupt protein folding (ex: C176F, H179R, C238S, C242S) [6]. The most well studied zinc-binding mutant is R175H, which happens to be the most frequently occurring missense p53 mutation in cancer [10]. The R175H mutation impairs zinc binding, resulting in misfolding of the protein, as well as loss of the ability to discriminate between consensus and non-consensus DNA sequence [27].

### 1.4. The Relationship between p53 Structure/Function and Zinc

The idea that the structure of a zinc-deficient mutant p53 protein could be restored to wild-type by reestablishing zinc binding is underscored by early research on the relationship of p53 to zinc. Specifically, the structure of p53 was found to be malleable by manipulating cellular zinc concentrations. In other words, p53 can be shifted to a misfolded form through the chelation of zinc, and this is reversible through supplementation of zinc [28]. Further, depletion of homeodomain-interacting protein kinase 2 (HIPK2), an activator of p53-mediated apoptosis and a negative regulator of metallothionein expression, results in misfolding of wild-type p53 and adoption of a “mutant-like” conformation [29,30]. Metallothioneins are a family of cytosolic zinc binding proteins, and their overexpression due to loss of HIPK2 leads to chelation of cellular zinc [31]. Zinc supplementation to HIPK2 depleted cells results in restoration of wild-type p53 structure and function, including consensus DNA-binding and transcription of p53-target genes [30,31]. Furthermore, in human cancer cell lines bearing p53-R175H (SKBR3) and R273H (U373MG), the mutant protein can be refolded through ZnCl_2_ supplementation [32]. Additionally, the refolded p53 gained the function of wild-type p53, including DNA binding and transcription of p53-target genes. 

### 1.5. Discovery of Thiosemicarbazones as Mutant p53 Reactivators

Zinc metallochaperones (ZMCs) were identified through a screen of the National Cancer Institute (NCI) database for substances that preferentially inhibited growth of human cancer cell lines expressing the hotspot *TP53* mutations at codons 175, 248, and 273 compared to cells expressing wild-type p53 [33]. The *in silico* approach identified several thiosemicarbazones that more strongly effected cells expressing one of the three hotspot mutations, but the compound NSC319726—later renamed ZMC1—was the most potent. ZMC1 induced apoptosis in a p53-R175H-dependent manner and induced a wild-type conformation change to the mutant protein. The wild-type conformation change coincided with an upregulation in the expression of p21, a p53-target gene. A critical observation was the transient nature of ZMC1 activity as measured by the levels of the mutant p53 protein. ZMC1 was able to inhibit the growth of xenograft mouse tumors derived from human cell lines expressing p53-R175H, but not tumors from cell lines expressing wild-type p53 or p53-R273H. 

The first clue that the mechanism of ZMC1 was zinc-dependent was revealed in the original publication identifying NSC319726. Thiosemicarbazones were already known to be chelators for iron, copper, and zinc [34], however, zinc is the only ion required for proper folding of p53 [26,27]. Supplementation of ZnCl_2_ within a narrow range (5–15 µM) was able to increase the cell growth inhibition activity of ZMC1 two-fold [33]. Another component to the mechanism was also revealed relating to the compound’s reactive oxygen species (ROS) properties. Thiosemicarbazones are known to chelate iron and cause oxidative stress by the production of hydroxyl radicals via Fenton chemistry [35]. To determine whether redox changes were an important component of the mechanism of ZMC1, they treated TOV112D cells with the reducing agent N-acetyl-cysteine (NAC) and found that it was capable of inhibiting the apoptotic activity of ZMC1. These were the first two findings that lead to the later discovery of the dual mechanism of ZMC1.

During the initial discovery of ZMC1, two other thiosemicarbazones were identified using the *in silico* screen [33]. NSC319725 (hereafter ZMC2) and NSC328784 (hereafter ZMC3) were characterized and compared to 3-aminopyridine-2-carboxaldehyde (3-AP or triapine) to determine if any other thiosemicarbazones functioned as zinc metallochaperones [36]. Triapine is a thiosemicarbazone that is being investigated in phase 2 clinical trials as an anti-cancer therapy for myeloproliferative neoplasms, advanced-stage cervical and vaginal cancers, and advanced non-small cell lung cancer [37,38] ZMC2 and ZMC3, but not triapine, exhibited zinc metallochaperone activity [36]. ZMC2 and ZMC3 bound zinc in a range suitable for p53-R175H reactivation, whereas triapine did not bind zinc. Furthermore, ZMC2 and ZMC3 demonstrated enhanced sensitivity in p53-R175H cell lines, but triapine did not [36]. Just as ZMC1 restored wild-type conformation and p53 transcriptional activity to mutant p53-R175H, ZMC2, and ZMC3 were capable of inducing the same changes, while triapine was not [36]. Taken together, these studies indicated that not all thiosemicarbazones function as zinc metallochaperones. 

Another thiosemicarbazone in clinical development is COTI-2, a molecule designed using a proprietary drug computational platform [39]. This molecule has been suggested to exhibit mutant p53 reactivating activity; however, it has shown broad anti-cancer activity in multiple human cancer cell lines and mouse xenograft models. Many of the cell lines tested against COTI-2 were sensitive at nanomolar concentrations [39]. COTI-2 was more effective at inhibiting human colorectal tumor cell proliferation than the approved therapies cetuximab and erlotinib. Similar results were seen for two human non-small cell lung cancer cell lines. Despite the remarkably broad sensitivity of COTI-2 against numerous human cancer cell lines, there is no proposed mechanism of action for this small molecule. It is unlikely to be a mutant-p53-specific therapy, as both wild-type and mutant p53-expressing cells exhibited sensitivity to the agent [39]. 

### 1.6. Elucidation of a Novel Mechanism of Action in Cancer Therapeutics 

The group hypothesized that NSC319726 restored wild-type structure and function to mutant p53-R175H by functioning to restore zinc binding to the mutant protein by donating zinc. This is a novel concept as no small molecule has ever been shown to induce a conformation change in the protein by restoring zinc binding. They called such a molecule a zinc metallochaperone (ZMC). This was based on a model of zinc-dependent folding and misfolding of p53, where a single molecule of Zn^2+^ needs to be bound to p53 in the DNA-binding domain (DBD) [27,40]. First it was discovered that ZMC1 complexes with Zn^2+^ in a 2:1 ratio [41]. Next, it was determined that ZMC1 does not directly bind p53-R175H, which was a surprise finding. That led to the hypothesis that perhaps the compound was raising the intracellular concentration of zinc high enough to overcome the zinc binding K_d_ of the p53-R175H. To demonstrate this they needed to measure the K_d_ of the p53-R175H, which had never been done before. During their attempts to quantify the binding affinity of Zn^2+^ to the DBD of mutant p53, they discovered that mutant p53-R175H contains two zinc binding sites within the protein’s DNA-binding domain [41]. K_d1_ refers to the strong, native ligation site (measured at approximately 2 nM) and K_d2_ refers to the weaker non-native site (estimated to be approximately ≥ 1 μM). This observation supported the notion that p53-R175H existed in the apo (zinc free) form within the cell. Using in vitro biophysical assays, the group demonstrated that ZMC1 was capable of donating zinc to p53-R175H and inducing a wild-type conformation change. This was important because this was direct evidence that the molecule was capable (with the addition of zinc) of inducing a conformation change. In contrast, evidence of such a change only existed as cellular data using conformation-specific antibodies where it could not be concluded that the drug directly induced a conformation change (perhaps some other protein(s) was involved).

The zinc metallochaperone activity of ZMC1 applies to other p53 missense mutations with impaired zinc binding. While the R175H mutation is both the most common missense p53 mutation in cancer and the most common zinc-binding mutant, other missense mutations fall into this category and are amenable to re-metallization with ZMCs. Cell lines expressing p53-C176F (SK-PN-DW) and p53-C238S (LN-18) had similar EC50 values to cell lines expressing p53-R175H (TOV112D), and cells expressing p53-C242S (H841) and p53-C242F (H1755) were markedly more sensitive to ZMC1. Taken together, this data expands the potential population of tumors that may be treated by a ZMC. 

ZMC1-induced redox activates the newly refolded p53-R175H, as measured by induction of p53-target genes and post-translational modifications (PTMs) of p53 (p53 phosphorylation at serines 15 and 46). Treatment with the reducing agent N-acetyl cysteine (NAC) did not prevent ZMC1 from refolding the mutant protein, but it did diminish PTMs of p53 and prevented the p53-dependent transcription of *p21* and *PUMA*. This demonstrated that another component to the mechanism involves the transactivation of the mutant protein. In other words, the same PTMs that serve to increase wild-type p53 signaling are employed by the compound upon a wild-type conformation change of the mutant protein. 

**Figure 1:**
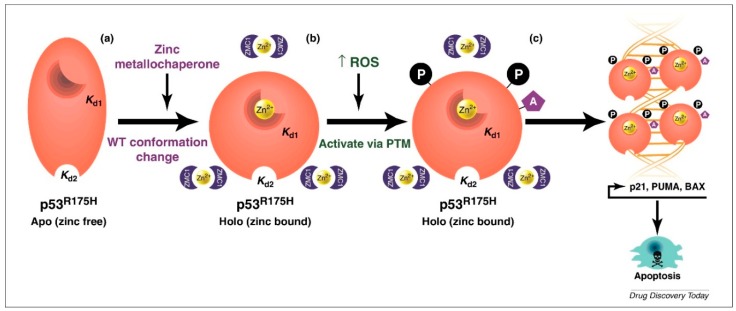
Model of the ZMC1 dual mechanism. (**a**) The p53^R175H^ is misfolded owing to impaired zinc binding (apo form) and, as a result, no site-specific DNA binding occurs (and no transcription of p53 targets). ZMC1 functions as a zinc metallochaperone (ZMC) by providing a source of zinc to facilitate refolding of the p53^R175H^ to a WT-like structure (holo form). (**b**) ZMC1 boosts reactive oxygen species (ROS) levels, which activates a stress response (i.e., ATM) that transactivates the p53^R175H^ through amino-terminal phosphorylation (P) and acetylation (A) events. (**c**) The newly conformed and transactivated p53^R175H^ can now bind DNA in a site-specific manner, causing transcription of apoptotic effectors that leads to tumor cell death. K_d1_ = K_d_ of the native binding site, K_d2_ = K_d_ of the non-native binding site.

In summary, the mechanism of ZMC1 is twofold and based on (1) zinc buffering to restore zinc binding and (2) induction of ROS to activate the newly refolded protein. Specifically, the K_d1_ of wildtype p53 is significantly lower than the K_d1_ or p53-R175H (~2 nM). The K_d2_ of both wild-type and mutant p53-R175H are ≥1 µM. At physiologic zinc concentrations, wild-type p53 is zinc bound (Holo), while p53-R175H is free of zinc (Apo). This explains why p53-R175H is misfolded under physiologic conditions, while wild-type p53 is folded. ZMC1 treatment increases intracellular zinc concentrations above 2 nM so that the R175H mutant binds zinc and re-folds. The second component of the ZMC1 mechanism is an induction of ROS, which triggers activation of p53 through PTMs (Figure 1 [42]). 

Further mechanistic research regarding the zinc ionophore properties of ZMC1 was described in 2015 [43]. Here it was demonstrated that ZMC1 raises intracellular zinc concentrations by moving extracellular zinc ions across the plasma membrane (ionophore function). A crystal structure of the compound in complex with zinc revealed a 2:1 binding stoichiometry in which the two hydrophobic backbones of ZMC1 face outwards, explaining how the 2:1 structure could pass through plasma membranes. This finding expanded the defining properties of ZMCs as mutant p53 reactivating molecules: (1) a ZMC must bind zinc with a greater affinity than the non-native zinc-binding site on p53 (K_d2_), but with an affinity lower than that of the native zinc-binding site (K_d1_); (2) a ZMC must be able to transport zinc across a plasma membrane. 

### 1.7. Pre-Clinical Translation of ZMCs

We have recently delineated how cellular zinc homeostatic mechanisms affect the pharmacodynamics of ZMCs [44]. Previously, it was shown that the pharmacodynamic activity of ZMC1 is transient, as reflected by the levels of mutant p53 and p21. Expression of the p21 protein rapidly increases after ZMC1 treatment, peaks at 6 h, and returns to baseline by 12 h. A similar pattern is seen for the kinetics of ZMC1-induced zinc influx. Zinc levels rapidly increase and peak at 4 h before returning to baseline by 8 h. Importantly, the transient increase in zinc precedes mutant p53 reactivation, and the activity of the drug (as measured by p21 levels) falls off just out of phase with the return to homeostatic zinc concentrations. 

We hypothesized that the transient nature of zinc influx was mediated by the cellular zinc homeostatic mechanisms. Cellular zinc levels are tightly regulated in eukaryotic cells, and significant deviations from basal levels can be toxic. As such, eukaryotes have evolved sophisticated zinc muffling mechanisms to respond to high free zinc levels. Three protein families, with 36 total members, are responsible for a coordinated response to changes in cellular zinc homeostasis–zinc importers (ZIP/SLC39 family, 14 members) [45], zinc exporters (ZnT/SLC30 family, 10 members) [46], and zinc binding proteins (metallothionein family, 12 members) [47]. Additionally, metal-responsive element-binding transcription factor-1 (MTF-1) is a zinc sensor that regulates the expression of zinc homeostatic genes [48]. We hypothesized that the ZMC1-induced zinc influx would trigger activation of these cellular zinc homeostatic mechanisms to counteract the increase in free zinc.

In response to ZMC1 treatment, cells have an increase in the expression of the exporter ZnT1 and the cytosolic zinc binding protein MT1A [44]. Simultaneously, there is a decrease in the expression of the zinc importer ZIP10. These three changes all function to counteract the zinc surge. Across the entire suite of zinc homeostatic genes, 16 of 37 genes displayed significant expression changes in response to ZMC1 treatment. *ZnT1*, *ZnT2*, *ZnT3*, *ZIP1*, *ZIP9*, *MT1G*, *MT1X*, and *MT2A* were markedly induced in TOV112D cells in response to ZMC1 treatment with kinetics that correlated with the ZMC1-induced zinc influx. *ZnT4*, *ZnT7*, *ZIP3*, *ZIP6*, *ZIP7*, *ZIP10*, *ZIP11*, *and ZIP14* had decreased expression in response to ZMC1-induced zinc influx [44].

After establishing that zinc homoeostatic genes are induced upon ZMC1 treatment, we sought to determine the functional relevance of the zinc homeostatic mechanisms in relation to ZMC1 pharmacodynamics. Using the CRISPR-cas9 genetic editing system, we developed a double knockout of *MT1A* and *MT2A* in the setting of the human ovarian cancer cell line TOV112D, which expresses p53-R175H [44]. 

The *MT1A/MT2A* KO cell line had greater and more prolonged increases in zinc in response to ZMC1 treatment [44]. Loss of expression of *MT1A* and *MT2A* impaired the cells’ ability to quickly respond to the zinc influx. The increased duration of elevated zinc levels corresponded with an increase in the duration and intensity of p21 expression, a marker of p53 reactivation. Likewise, the *MT1A/MT2A* KO cells were approximately three times more sensitive to ZMC1 than the parental cells. 

**Figure 2:**
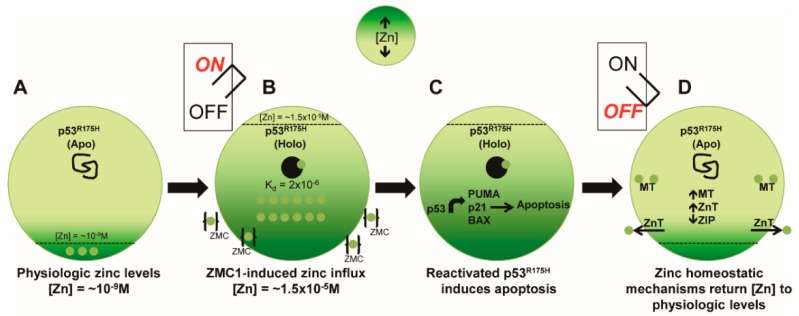
The ZMC Switch mechanism. (**A**) At physiologic zinc levels (10^−9^ M), p53^R175H^ is in its Apo (zinc free) state because the mutation weakens its binding at the native ligation site (p53^R175H^ Zn K_d2_ × 10^−6^). This causes the protein to misfold and lose its wild-type transcriptional function. (**B**) Upon treatment with a ZMC, intracellular zinc levels increase approximately 1000 fold (1.5 × 10^−5^ M), and this allows zinc to bind in the p53^R175H^ native ligation site and the protein adopts a wild-type conformation (on switch). (**C**) Once the protein undergoes a wild-type conformation change, the p53^R175H^ exhibits wild-type transcriptional activity and an apoptotic mechanism is induced. (**D**) Within hours the cell responds to this zinc surge by activating zinc homeostatic mechanisms (increasing expression of metallothioneins, zinc exporters, decreasing the expression of zinc importers) that function to lower cellular zinc levels to physiologic range. Zinc is no longer at a sufficient concentration to remain bound in the p53R175H and the protein returns to its Apo state (off switch).

Taken together, this data suggests an “on/off” mechanism of ZMC regulation in the cell. These data regarding the cellular response to zinc combined with the previous mechanistic research that was performed shaped a more complete and most up-to-date understanding of the mechanism of action of ZMCs. In a cancer cell expressing p53-R175H, a zinc-deficient mutation of p53, Zn^2+^ levels are kept in the picomolar range (10^−9^ M). It was previously determined that the zinc K_d_ of p53-R175H to be approximately 2 × 10^−6^ M [41], which would explain why the mutant protein is in its Apo (zinc free) form and is misfolded (Figure 2A). Treatment with a ZMC causes an influx of Zn^2+^ ions, raising intracellular zinc 1000-fold to approximately 1.5 × 10^−5^ M, which is sufficiently high enough to allow zinc binding in the native ligation site of p53-R175H, resulting in a wild-type conformation change (Holo form). The zinc surge and conformation change of mutant p53 is the ON switch that allows the newly refolded protein to function like wild-type p53 to induce apoptosis (Figure 2B,C). In response to the massive zinc surge, cellular zinc homeostatic mechanisms are activated to return zinc levels back to physiologically appropriate levels. This includes upregulation of MT and ZnT genes that decrease intracellular zinc, and downregulation of ZIP genes that serve to increase intracellular zinc. As a result of the cellular zinc homeostatic response, zinc levels decrease and zinc can no longer bind to p53-R175H, forcing the mutant protein to return to its Apo (zinc free) form, effectively turning the switch off (Figure 2D). 

This on/off switch mechanism is unique in cancer therapeutics and the in vivo translation suggests that ZMCs might not need maximal or continuous dosing for optimal efficacy. If the activity is turned off within hours, then perhaps the exposure required to produce this activity need only be brief. Through a series of assays, it was discovered that short exposures of ZMC1 (as short as 15 min) were capable of preventing growth in a colony formation assay, and that short exposures were able to induce expression of the p53-target gene p21 [44]. 

Pre-clinical studies of ZMC1 have recently been performed using the genetically engineered KPC mouse model of pancreatic cancer (Pdx-1-Cre; Kras^G12D/+^; p53^R172H/+^, or p53^R270H/+^) in which the concept of the switch mechanism has been translated in vivo [44]. In this model p53^R172H/+^ is a zinc deficient allele (mouse homologue of the human p53R175H and positive control), and the p53^R270H/+^ is non-zinc deficient allele (mouse homologue of the p53R270H, negative control). The pharmacokinetic studies revealed that ZMC1 reached a C_max_ of 2.4 μM (well above the concentration necessary for mutant p53 reactivation) with an AUC_last_ of 0.385 h × μM and a half-life of 0.59 h which indicates that the compound is cleared rapidly [44]. This pharmacokinetic profile would normally be disadvantageous for a traditional targeted therapeutic; however, for ZMC’s this turned out to be an advantage. The duration of exposure is sufficient for the on/off switch mechanism to occur and this was supported by several lines of evidence: (1) pharmacodynamic experiments in which p53 downstream targets and markers of apoptosis were increased upon ZMC1 treatment in the KPC-p53^R172H^ as compared to the KPC-p53^R270H^ and (2) ZMC1 as a single agent significantly increased the survival of the KPC-p53^R172H^ mice while having no effect on the KPC-p53^R270H^ [44]. 

### 1.8. ZMCs Synthesized in Complex with Zinc: A Novel Drug Formulation

Recently we have synthesized a novel formulation of ZMC1 in which the monomer is synthesized in complex with zinc in a 2:1 molar ratio [44]. We have termed the complex Zn-1. We hypothesized that the complex would improve zinc delivery and, thus, be more potent. Cell growth inhibition assays showed than Zn-1 was in fact more potent than ZMC1. Using LC/MS/MS we were able to detect both the complex (Zn-1) and its monomeric units (ZMC1) in the serum of Zn-1-treated mice. In KPC-p53^R172H^ mice, Zn-1 was more potent and less toxic than ZMC1. Likewise, Zn-1 treatment increased the survival of the KPC-p53^R172H^ mice both compared to vehicle control (*p* = 0.00065) and the ZMC1 monomer (*p* = 0.023). 

### 1.9. Pharmacokinetic Parameters That Distinguish ZMCs

This on/off switch has important implications for the translation of ZMCs to the clinic. While most targeted cancer therapeutics are designed to specifically bind a molecular target for as long of a duration as possible (i.e., kinase inhibitors and receptor blockers), ZMCs do not bind their molecular target (mutant p53) at all. Moreover, the traditional paradigm in targeted drug development is to optimize compounds through medicinal chemistry for pharmacokinetic properties (i.e., T_1/2_, AUC_max_) that maximize drug exposure to ensure the molecule is bound to its target as long as possible. All ZMCs have some affinity for other divalent metal ions including the chelation of redox active iron and copper, which is a source of toxicity. The on/off switch of ZMCs is a considerable advantage because it allows for brief exposures that minimize toxicity. From a pharmacokinetic perspective, ZMCs need not be optimized for maximal exposure, but rather to achieve the optimal maximum concentration (C_max_) in serum to increase intracellular zinc concentrations sufficiently to reactivate mutant p53 (above the EC_50_ of the tumor cell). 

### 1.10. Design Principles for Future Clinical Trials of a ZMC

ZMCs differ significantly from standard and targeted therapeutics on several levels from the specificity of p53 mutants, to the way they engage their target, to their transient pulsatile kinetics, and their pharmacokinetic profile. All of these will need to be considered when designing the first in-human clinical trial. 

#### 1.10.1. Patient Selection

Patients will need to be sequenced to determine the p53 status of their tumors and whether they harbor a zinc-deficient mutation. R175H will likely be the most common mutation in the cohort, as it is the most common p53 missense mutation, but other mutations will be included to broaden the potential pool of patients. While our research has expanded the spectrum of zinc deficient p53 mutants [41], the full spectrum is unknown. We hypothesize that a number of other missense mutants within close proximity to the zinc-binding pocket may also impair the protein’s ability to bind zinc. Further research is needed to define the spectrum of missense mutants that can be reactivated by ZMCs. 

#### 1.10.2. Proof-of-Concept Clinical Trial 

In today’s highly competitive world of targeted drug development, there is a heightened need to understand not only whether a particular drug is safe but, more importantly, if there is evidence that it can hit its target. Thus, proof-of-concept clinical trials are taking the lead in early phase clinical development. We feel this should also apply to the first in-human clinical trial with a ZMC. While most phase I trials employ a traditional dose escalation study to determine the maximum tolerated dose (MTD), for a ZMC the MTD is not necessary and will induce off-target toxicity. Dose modulations will be guided by their C_max_ levels and the endpoint will be a corresponding assay of mutant p53 reactivation in the tumor. We have found that measuring p53 transcriptional targets as a biomarker of activity is challenging, and that markers of apoptosis are more optimal. Additional study endpoints would include reduction in tumor size and disease-free survival. 

#### 1.10.3. Biomarkers of Sensitivity/Resistance 

As with any cancer therapeutic, there will be responders and non-responders. We have found ([49]) a mechanism of innate resistance to ZMCs in the dysregulation of expression of zinc homeostatic genes. Numerous studies have described dysregulation of zinc homeostatic genes in breast [50], pancreatic [51], and prostate cancer [52]. Cancer cells that express abnormally high levels of genes that function to lower zinc levels (such as metallothioneins) will likely impair a ZMC’s ability to raise zinc levels sufficiently high enough to reactivate mutant p53. Thus, for now, it seems that high metallothionein expression is a biomarker of resistance to ZMCs. In future clinical trials, it is likely that patients will also be screened for the full suite of zinc homeostatic genes as transcriptomic profiling becomes more widely employed. 

## 2. Conclusions

Zinc metallochaperones represent a new pharmacological approach to the reactivation of zinc-deficient mutant p53 proteins. Significant progress has been made in the understanding of the mechanism of ZMCs indicating some very unique properties in the field of cancer therapeutics. Pre-clinical evidence now exists that demonstrates how these properties can be translated in vivo. Further research is now needed to optimize ZMC1 for drug-like properties to find a molecule that can be introduced into the clinic. Recently we have also innovated the formulation of ZMC1 by synthesizing it in complex with zinc in a 2:1 ratio [44]. Pre-clinical evidence using a genetically engineered murine model of pancreatic cancer shows that this formulation has greater efficacy than ZMC1. This suggests that the future clinical candidate ZMC should also be synthesized as a drug:zinc complex. One of the most attractive aspects of developing ZMCs as anti-cancer drugs is that the patient population in whom to treat is theoretically known, which is also unusual in cancer therapeutics. Further research will need to be done to determine the effects of chronically pulsing zinc in patients. Would acquire resistance such as selecting for a non-zinc deficient p53 mutation occur over time with exposure to a ZMC? Would ZMCs increase the effectiveness of cytotoxic chemotherapy or radiation, given that both activate wild-type p53? If the toxicity of ZMC1 is due to the chelation of other redox active divalent metals (Fe^2+^, Cu^2+^), could assays be developed to measure iron and copper binding to facilitate the design of a compound that better preferentially binds zinc while having a weaker affinity for iron or copper? These questions will likely be answered the coming several years.
